# Moderate Hydronephrosis among Acute Ureteral Calculus on Ultrasonographic Imaging in a Tertiary Care Center in Nepal: A Descriptive Cross-sectional Study

**DOI:** 10.31729/jnma.6879

**Published:** 2021-12-31

**Authors:** Dipesh Paudel, Deepak Adhikari, Radha Devi Dhakal

**Affiliations:** 1 Department of Radiodiagnosis and Imaging, Chitwan Medical College Teaching Hospital, Bharatpur, Nepal; 2 Department of Nursing Faculty, Shree Medical and Technical College, Bharatpur, Nepal

**Keywords:** *hydronephrosis*, *ultrasonography*, *ureteral calculus*

## Abstract

**Introduction:**

Ureteric calculi are lying at any point of ureter from the pelvic ureteric junction to the vesicoureteral junction. If left untreated, ureteropelvic junction obstruction can lead to hydronephrosis. With the improved availability of computed tomography and ultrasound scanning, hydronephrosis is being diagnosed more frequently. The main aim of this study is to find out the prevalence of moderate Hydronephrosis among ureteral calculus on ultrasonography imaging in a tertiary care center of Nepal.

**Methods:**

A descriptive cross-sectional study was conducted among 110 acute ureteral calculus cases at Radiodiagnosis and Imaging Department of Chitwan Medical College and Teaching Hospital (CMCTH), Bharatpur from 15th August 2020 to 15th May 2021. The ethical approval was taken from the Institutional Review Committee of same institution. Convenient sampling technique was used to select the participant. The collected data was entered in excel 16 and analysed in Statistical Package for Social Sciences. Point estimate at 95% Confidence Interval was done and frequency and percentage were calculated.

**Results:**

Out of the 110 cases of acute ureteral calculus, 31 (28.2%) (19.79-36.60 at 95% Confidence Interval) has moderate hydronephrosis in the ultrasonographic imaging. The mean age of participants was 31.61±8.51 years and male to female ratio was 1.97:1. Vesicoureteric junction was the most common site for ureteric calculus 39 (35.5%).

**Conclusions:**

The ultrasound is an easy method to be applied, and a fast one to help and diagnose obstructive hydronephrosis. The main causes of hydronephrosis are kidney stones, followed by ureteral stones, with a moderate degree of hydronephrosis.

## INTRODUCTION

Hydronephrosis is dilatation of the pelvicalyceal system due to kidney stones, followed by ureteral stones, in which, in a larger percentage detected on ultrasonography.^1-5^ Patient present with flank pain, abdominal mass, urinary tract infection, fever, painful urination (dysuria), increased urinary frequency, increased urinary urgency, etc.^6^

The obstructive hydronephrosis is a term that implicates the structural and functional changes of the kidneys as a result of difficulties in the flow of urine (difficulties in urinating).^7^ Using an ultrasound-first approach to detect hydronephrosis may help physicians identify patients with renal colic.^8^

The main aim of this study is to find out the prevalence of moderate hydronephrosis among ureteral calculus on ultrasonography imaging in a tertiary care center of Nepal.

## METHODS

A descriptive cross sectional study design was undertaken at Radiodiagnosis and Imaging Department of Chitwan Medical College and Teaching Hospital (CMCTH), Bharatpur from 15th August 2020 to 15th May 2021. Study population were those adult patients with ultrasound findings of acute ureteral calculus were eligible for enrolment for the study. Chronic ureteric calculus, follow up cases of ureteric calculus, children, and pregnant woman with uretic calculus were excluded in this study.

n = Z^2^ × p × q / e^2^

  = (1.96) ^2^ × 0.5 × (1-0.5) / (0.1)^2^

  = 0.9604/0.01

  = 96.04

Where,

n= required sample size
Z= 1.96 at 95% Confidence Interval (CI)
p= prevalence 50% for maximum sample size
q= 1-p
e= margin of error, 10%

Hence, the calculated sample size was 96.04. Taking a 10% non-response rate and rounding the figure, we included 106 patients in the study. However 110 samples were included in the study.

Final required sample size was 110. Convenient sampling technique was used to select the participant. Prior to data collection ethical approval was obtained from the Institutional Review Committee CMC-IRC/077/078-014 and written informed consent was taken from participants. Researcher himself collected the data. Face to face interview and ultrasound machine were used to collect the information. The ultrasound was performed using SAMSUNG MEDISON HS70 AND HS 40 systems with a CH5-2 convex transducer) by two radiologists with more than five years' experience. All the patients were examined in moderately full bladder no other special preparation were needed prior to ultrasonogram. Patients were examined in supine position, right lateral, left lateral and prone positions as required. All the areas for calculus location was closely examined from renal pelvis, ureter (upper, mid and lower), the vesicoureteral junction (VUJ) and bladder. Maximum diameter was recorded in any plane for the size of the stone. Number of stones were counted. Most of the cases presented with hydro ureteronephrosis so grading was done on the basis of the proximal dilatation of Ureter. Greater the grade of the hydro ureteronephrosis, more severity of the ureteral obstruction was consider. Definitive diagnosis of calculus was done if there were clear visualization of the echogenic focus within the ureter followed by proximal dilatation of the ureter and twinkling artefact on Doppler imaging. More manual pressure was applied on the probe to displace the intestine to reduce gas interference during the examination. Time taken for the examination was variable, 3 to 6 minutes depends upon bowel gas and obese patient. Accuracy of ultrasound machines were obtained and maintained by using same machine and measurement scale for all the respondents.

Collected data were checked for accuracy and completeness. Data was entered in excel 16, analysed in Statistical Package for the Social Sciences (SPSS) 20 version and interpreted by using descriptive statistics method in terms of frequency, mean, percentage. Point estimate at 95% Confidence Interval was done and frequency and percentage were calculated.

## RESULTS

Out of the 110 cases of acute ureteral calculus, 31 (28.2%) (19.79-36.60 at 95% Confidence Interval) has moderate hydronephrosis on ultrasonographic findings. Among the cases of moderate hydronephrosis, the size of calculus was >5mm in 27 (45.80%) cases and ≤5mm in 4 (7.80%) cases. The calculus found in distal, proximal, mid ureter and ureteropelvic junctions are 10 (37%), 8 (38.10%), 7 (28.00%), and 5 (12.50%) respectively (Table 1).

**Table 1:** Features according to the grades of hydronephrosis.

Characteristics	Grade of Hydronephrosis n (%)	
Size of calculus	Mild	Moderate
≤5	47 (92.20)	4 (7.80)
>5	32 (54.20)	27 (45.80)
Calculus in proximal ureter	13 (61.90)	8 (38.10)
Calculus in mid ureter	18 (72.00)	7 (28.00)
Calculus in distal ureter	17 (63)	10 (37)
Calculus in ureteropelvic junction	35 (87.50)	5 (12.50)

Out of the 110 cases of ureteral calculus, the majority 29 (26.4%) of the participants were in the age group of 26-30years and 7 (6.4%) were in the age group of 41-45years. The mean age of participants was 31.61 ±8.51 years (minimum = 17 and maximum = 52years) (Table 2). Most of the cases were male 73 (66.4%) and female were 37 (33.6%).

**Table 2:** Demographic features of the patient (n = 110).

Characteristics	Category	n (%)
**Age in group**	<20	7 (6.4)
	20-25	20 (18.2)
	26-30	29 (26.4)
	31-35	21 (19.1)
	36-40	16 (14.5)
	41-45	7 (6.4)
	>45	10 (9.1)
**Sex**		
	Male	73 (66.4)
	Female	37 (33.6)

Right kidney was mostly affected 59 (53.6%), and mostly the hydronephrosis was of mild grade 79 (71.8%). Vesicoureteric junction was the most common site for ureteric calculus 39 (35.5%) and mostly the size was between 2.5-5mm.

**Table 3:** Size of ureteric calculus and grades of hydronephrosis.

n (%)		
Affected kidney side	Right side	59 (53.6)
	Left side	51 (46.4)
Grade of hydronephrosis	Mild	79 (71.8)
	Moderate	31 (28.2)
Location of ureteric calculus	Proximal ureter	18 (16.4)
	Mid ureter	26 (23.6)
	Distal ureter	27 (24.5)
	Vesico ureteric junction	39 (35.5)
Size of calculus (mm)	2.5-5	51 (46.4)
	5.1-7.5	45 (40.9)
	7.6-10	14 (12.7)
**Mean±SD = 5.67±1.46**		
**Small = 2.5mm, Large = 10mm**		

## DISCUSSION

Ultrasound should be used as the primary diagnostic imaging tool.^9^ Widespread use of ultrasound imaging for diagnosis of renal problems has led to an increased rate of diagnosis of renal calculus with hydronephrosis in the last decades.^10^ In this study, we focused on the ability of ultrasound imaging to determine the urinary stones in the urinary tract with grades of hydronephrosis. We selected 110 cases with acute ureteric calculus, all were presented with hydronephrosis. Only two grade hydronephrosis graduate mild and moderate, were found during our sonographic examination. In this study ureteric calculus with hydronephrosis is most commonly presented to men with an average age about fifties. We came to know that there is a significant relationship between detection of calculus and the grade of hydronephrosis. Increasing the size of calculus increases the grade of hydronephrosis.

In this study most of the patient were male (male 66.4% vs female 33.6%) and most affected kidney is right sided 53.6% vs left 46.4. This findings are supported by the findings of study conducted by Sultan, et al.^3^ where males account for 67.14% of the sample and female 32.86%. The right kidney affected more than the left (52.7% vs. 41.4%). In this study majority 26.4% of the participants were in the age group of 26-30 years with mean age was 31.61. This finding is in the line of the study findings of Kaplon, et al.^11^ where most of the patients present aged between 30 and 60 years of age, with peak incidence between 35-45 years old. Average age of presentation is 33.4 years in the study findings of Joshi, et al.^12^ males are more likely to suffer from urinary calculi.^13^ The lifetime prevalence of ureteric calculi is relatively high, occurring in approximately 12% of men and 7% of women.^14^ even an individual's risk will vary greatly throughout the life, If an individual has a history of stone disease then their future risk of recurrence is high.^15^

In this study common ureteral calculus location was found in upper ureteric junction 35.6%, 16.4% found at proximal ureter. This finding is supported by the study of Song, et al.^16^ which revealed that calculus location in vesico ureteric junction was 47.1% and proximal ureter was 21%. Usually medium and large renal lithiasis (> 5mm) can be easily detected with 2D ultrasonography due to the different echogenicity with the adjacent parenchyma and the posterior acoustic shadowing.^17^ In the other hand small renal lithiasis less than 5mm, differential diagnosis between suspected renal lithiasis and hyperechoic foci caused by other factors (e.g. vascular and/or parenchymal calcifications, clots, arcuate arteries) is difficult.^18^ In this study regarding size of calculus the smallest calculus detected was 2.5mm and the largest calculus was 10mm, average size was 5.67. This finding is contrast with the findings of Joshi, et al.^12^ where out of 256 examining the size of calculus, 35 calculi were small <5mm; 207calculi were medium (5-15mm) and 11 were large > 15mm. The average size of the calculus is 7.8mm. This might be due to the difference in sample size and data collection duration . We found the cases only from mild and moderate grade hydronephrosis.

In a similar article, Chi, et al. urolithiasis is the most common cause of hydronephrosis in the adult patient and has a prevalence of 10%-15%.^19^ The detection rate of calculus increases with increasing the grade of hydronephrosis from grade-1 to grade-3 then drops in Grade-IV in Alshoabi, et al.^12^ Ultrasound-detected hydronephrosis was present in approximately 90% of patients with urinary stones Moak, et al.^20^

This study was confined to only one center with a limited sample size. A larger scale, multi-centre study with a more diverse population needs to be conducted to minimize the bias and establish more generalizable findings of the problem.

## CONCLUSIONS

Ultrasound can be used as the primary diagnostic imaging tool in suspected cases of acute ureteric calculus. Urolithiasis is the most common cause of hydronephrosis. The detection rate of calculus increases with increasing the grade of hydronephrosis.

Future studies regarding ultra-sonographic evaluation of ureteric calculus with follow-up prospective study and comparative study on Ultrasonography versus Computed Tomography for Suspected renal calculus can be conducted.
